# Growth of Optically Active Chiral Inorganic Films through DNA Self-Assembly and Silica Mineralisation

**DOI:** 10.1038/srep04866

**Published:** 2014-05-02

**Authors:** Ben Liu, Lu Han, Yingying Duan, Yunayuan Cao, Ji Feng, Yuan Yao, Shunai Che

**Affiliations:** 1 School of Chemistry and Chemical Engineering, State Key Laboratory of Metal Matrix Composites, Shanghai Jiao Tong University, 800 Dongchuan Road, Shanghai, 200240, China

## Abstract

The circularly polarized reflection of nature is due to their distinct azimuthally twisted or helical character in the nanostructure of the surface films. Although many chiral inorganic powders have been successfully synthesised, the artificial synthesis of chiral inorganic films is rare. Herein, we reported a facile synthetic route for the growth of monolayered chiral film on the quaternary ammonium-modified silicon substrate. The films grew on the substrate surface because of the strong electrostatic interaction between positively charged quaternary ammonium groups and negatively charged phosphate groups of DNA, with subsequent growth to right-handed, vertically aligned, impeller-like helical architectures with left-handed two-dimensional square *p*4*mm-*structured DNA chiral packing. The DNA–silica composite films exhibited strong optical activity at 295 nm and in the range of 400–800 nm, corresponding to DNA chiral packing (absorption) and to the helical blade in the impeller (scattering), respectively. Upon removal of DNA templates, the pure inorganic impeller-like helical morphology was maintained; consequently, the scattering-based optical response was blue-shifted approximately 200 nm as a result of a decrease in the effective average refractive index. The hierarchical structures were reflected from the surfaces by cross-polarised light, which confirmed that the films were strongly birefringent, with long-range anisotropy.

Chirality is a basic attribute of nature and can be found in objects ranging from the microscopic level (*e.g.*, amino acids, DNA and proteins) to the macroscopic level (*e.g.*, sea snails, trees and even the universe)^1^,^2^,^3^. The visual appearance of many animals is well known to be determined by structural processes and not by pigments^4^,^5^. The circularly polarised reflection from scarab beetles (*i.e.*, *Plusiotis gloriosa*) is due to their distinct azimuthally twisted or helical character in the nanostructure of the films^6^. Chirality is ubiquitous in organic materials with helical geometries. Therefore, many organic chiral films have been extensively investigated for use in electronic and optoelectronic applications^7^,^8^,^9^. Optically active chiral inorganic materials have also been extensively investigated for use in catalysis, separation, electronic and optoelectronic applications because of their inherent electronic, optical and mechanical properties and their thermal stability^10^,^11^,^12^,^13^,^14^. One of the most promising states of these materials is the film form, which is desirable for application in electronic and optoelectronic devices for mimicking functional biosystems. However, only three methods have been reported for the successful artificial synthesis of chiral inorganic films: MgF_2_ films have been prepared using engineered glancing-angle deposition (GLAD)^15^; a sculptured chiral thin film has been formed using the serial bideposition (SBD) technique^16^; and free-standing helical mesoporous silica films^17^ and derived materials^18^,^19^,^20^ have been prepared through the self-assembly of nanocrystalline cellulose (NCC).

From a technological point of view, the preparation of chiral inorganic solids in the form of thin helical films on supporting substrates via the self-assembly of chiral organic templates and inorganic sources is desirable. However, attempts to achieve this goal have been largely unsuccessful because the interaction between the chiral templates and the surface (the supporting substrate) must be built up and because the subsequent chiral co-assembly processes of templates and inorganic precursors on the substrates must be controlled.

The strategy that we used to synthesise chiral silica films relied on controlling the growth of helical DNA–silica impeller^21^,^22^ on the substrate surface by facilitating the interaction between the DNA and the substrate and their subsequent self-assembly with a silica source^23^. A Si (100) wafer was used as the supporting substrate (see Supplementary Figure S1 and Supplementary Figure S2 a_1_, b_1_). The sonicated B-DNA, which ranged in length from 100 to 300 bp (which approximately corresponds to the persistence length of the molecule), has been used in this synthesis (Supplementary Figure S3). The procedure for the direct growth with helical inorganic morphology on the substrate surface was separated into five preparation steps (Figure 1): (i) The silicon substrate for containing the silanol groups on the surface were first cleaned in sequence with HF acid solution, acetone, ethanol and deionised water three times (Figure 1A). The silicon substrate was also pretreated with H_2_SO_4_/H_2_O_2_ to increase the abundance of silanol groups^24^. (ii) The chemical modification of a positively charged quaternary ammonium functionalised surface via the silanisation of silanol-based surfaces using quaternary aminosilane (*N*-trimethoxysilylpropyl-*N,N,N*-trimethylammonium chloride, TMAPS) to terminate the substrate surface was employed (Figure 1B)^24^. (iii) The DNA layer was introduced onto the substrate surface via strong electrostatic interactions between the positively charged quaternary ammonium groups on the substrate surface and the negatively charged phosphate groups of DNA (Figure 1C). (iv) The synthesis gel mixture composed of DNA, MgCl_2_, TMAPS, TEOS and H_2_O was subsequently introduced to induce growth of the chiral DNA–silica films (CDSFs) on the substrate. In this process, TMAPS acts as both a condensing agent and a co-structure-directing agent (CSDA)^13^ and results in the self-assembly of the DNA–silica composite with co-condensation of the silane group of TMAPS and the silica source of tetraethoxysilane (TEOS), and Mg^2+^ acts as an inducer of DNA chiral packing^21^ of the chiral morphological particles in the DNA–silica composite (Figure 1D). (v) We calcined the CDSFs to pure inorganic chiral silica films (CSFs) by removing the DNA templates (Figure 1E).

The CDSFs give rise to dual optical activity (OA): (i) absorption-based OA arising from DNA chiral packing, *i.e.*, an optical response to circularly polarised light (CPL) at the absorption bands due to transition dipole moments between the chromophores of DNA in close proximity of the long-range chiral DNA packing (see Supplementary Figure S4 and S5)^21^,^25^,^26^,^27^,^28^; (ii) scattering-based OA arising from helical architecture with a periodic pitch length where at the circular Bragg resonance, the film would reflect one handed CPL and another one handed light would be transmitted and subsequently absorbed by the silicon substrate^15^,^29^,^30^,^31^.

## Results

### Chiral morphologies and strucutres of the films

**Figure 2:**
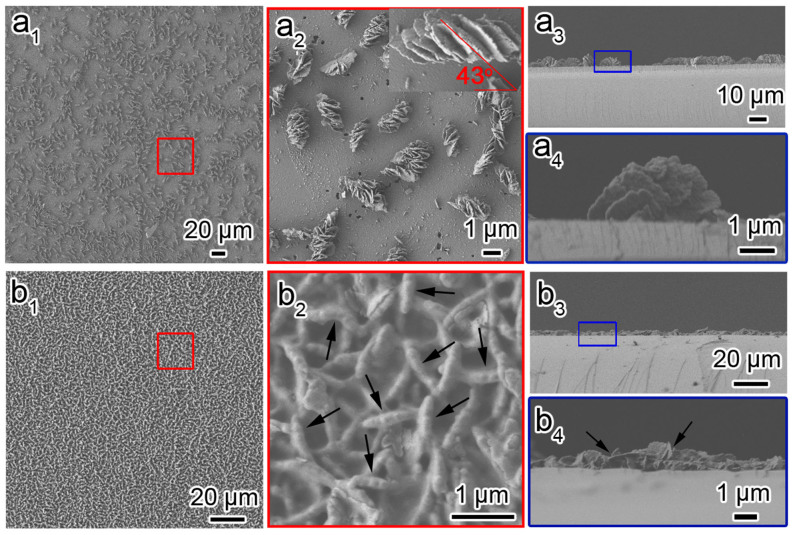
Macroscopic helical morphologies of CDSFs. Top view and side view of the low- and high-magnification SEM images of CDSFs formed on the silicon substrates without pretreatment (a_1–4_) and with H_2_SO_4_/H_2_O_2_ pretreatment (b_1–4_) showing the impeller-like helical architecture composed of several blades grown on the substrate. The molar composition of the synthesis gel was DNA (phosphate group):Mg^2+^:TMAPS:TEOS:H_2_O = 1:1.5:6:15:18 000.

Both the as-prepared CDSFs and the calcined CSFs were macroscopically smooth and glossy with the same appearance as a pure silicon wafer, which indicated that thin and uniform films can be formed on the silicon substrate (see Supplementary Figure S2). Figure 2 shows the scanning electron microscopy (SEM) images of the as-prepared CDSFs synthesised without and with the surface pretreatment. As shown in Figure 2a1–4, the film synthesised without the surface pretreatment was composed of particles with impeller-like helical morphology, which is evident in the incline-arranged blades from the top view and the half-impeller from the side view. The half-impeller had a uniform radius of ~2 μm and a uniform thickness of ~100 nm (*i.e.*, the thickness of the blades corresponding to the length of DNA molecules). The blades of the vertically aligned impeller were stacked in a single/uniform direction, which revealed unambiguously the handedness of the macroscopic helical morphology. The impeller-like helical DNA–silica complex (IHDSC) with blades arranged in an anticlockwise manner in the side view (*i.e.*, at an inclination angle smaller than 90° from right-bottom to left-top from the top view as shown insert in Figure 2a2) is defined as right-handed, and the opposite case (see Supplementary Figure S6) is defined as left-handed. We estimated the enantiopurity by counting the characteristic morphologies of more than 1000 randomly chosen particles in the SEM images, which were obtained from more than 20 different regions of the film surface according to the inclination angle of the blades. The CDSFs synthesised without the surface pretreatment were found to be predominantly right-handed with an absolute enantiomeric excess (*ee*) of approximately −90% (*ee* value was defined as 100% × [(l − r)/(l + r)], where l and r are the amounts of the left-handed and right-handed helical impellers in the given sample). The blades had similar inclination angles in the range of 36 to 46° (insert in Figure 2a2), and the corresponding pitch lengths of the helical blades were calculated to be 20–15 μm. The number of blades in each half-impeller ranged from 6 to 10.

**Figure 3:**
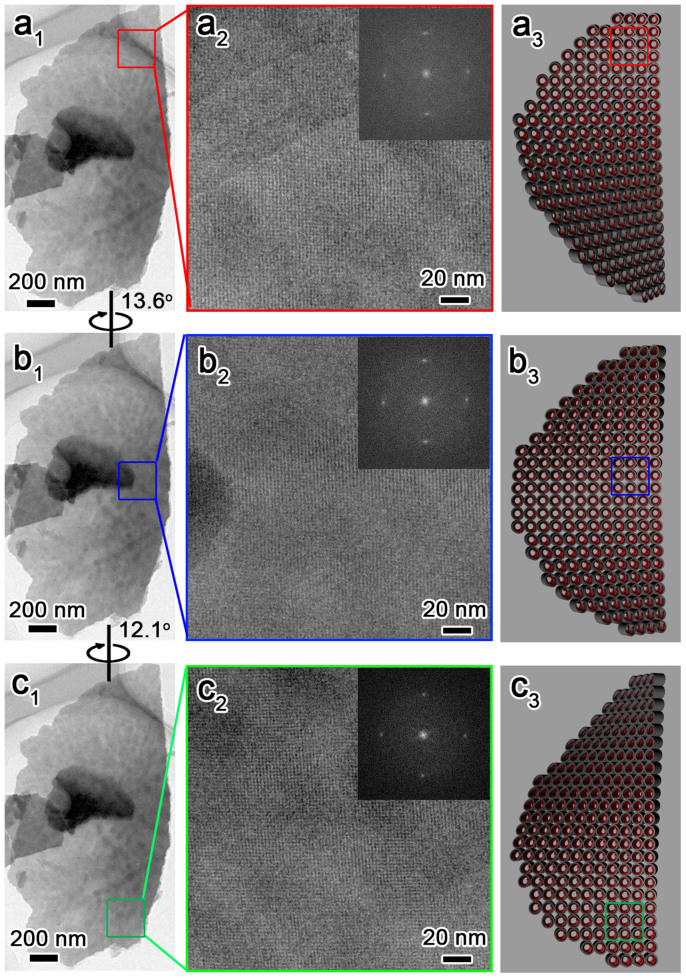
Microscopic helical structures of DNA chiral packing, macroscopic helical morphologies of the twisted blades and the corresponding schematic representation of the CDSFs shown in Figure 1a. The blades were bent, and the 2D-square *p*4*mm* lattice aligned with the incident electron beam in the top region (a_1_–a_3_). We aligned the middle (b_1_–b_3_) and bottom (c_1_–c_3_) portions by tilting the sample in a clockwise manner along its (10) axes by 13.6° and then 12.1°, which unambiguously indicated left-handed DNA chiral packing and left-handed helical blades.

**Figure 4:**
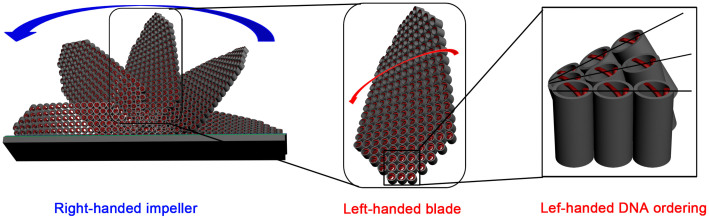
Schematic representation of a multihelix of CDSFs. The right-handed impeller contains left-handed twisted blades and left-handed chiral DNA packing.

The intrinsic chiral nature of the CDSFs, such as the helicity of the blades and especially the handedness of the DNA packing in the helical blades, has been determined from detailed high-resolution transmission electron microscopy (HRTEM) observations (Figure 3). We prepared the sample by mechanically removing (scraping) some impellers and blades from the substrate; the impellers and blades were dispersed in ethanol and collected for HRTEM observation. The blades with a highly ordered two-dimensional (2D)-square *p*4*mm* mesostructure^21^,^32^ and a unit-cell parameter of *a* = 2.5 nm were bent along the (01) plane and were only aligned with the incident electron beam in a local region, which indicated a long-range chiral arrangement (Figure 3). It showed a 1D misaligned fringe along the middle of blades and finally became a completely misaligned contrast at the bottom of the blades. We could align the 2D-square contrast in the middle and bottom portions by tilting the blades clockwise along the (10) axis by 13.6° and 12.1°, which indicated that the twisted DNA columnar packing in the CDSFs is left-handed (Figure 4)^21^. The distances from the top to the middle portion (0–13.6°) and from the middle to the bottom portion (13.6–25.7°) were ~0.74 and ~0.69 μm, respectively. Therefore, the average pitch length of the blades was calculated as (0.74 + 0.69 μm) × 360°/25.7° = 20 μm, and the average tilting angle per DNA layer was calculated as (2.5 nm × 360°)/20, 000 nm = 0.045°. These results are consistent with the results calculated for the helical blades from the inclination angles. On the basis of the previously described microscopic and macroscopic observations, we concluded that the DNAs have left-handed long-range chiral packing in the right-handed impeller (*i.e.*, the right-handed impeller is constructed of left-handed blades) (Figure 4). The inclination angles and corresponding pitch lengths of the helical impeller were easily tuned as functions of the amount of DNA chiral packing inducer (Mg^2+^)^33^. The remarkable inclination angle transition from 0° to 16° to 68° corresponds to a decrease in pitch length from infinite to 45 to 10 μm (see Supplementary Figure S7). The 2D-square *p*4*mm* structure was formed as a result of the electrostatic “zippers” interaction between the quaternary ammonium groups of TMAPS and the phosphate groups of DNA with exceptionally small interaxial separation^32^. The macroscopic morphological evolution revealed that the impeller-like helical architectures were formed from DNA–silica platelets erected on the substrate surface to the breakage of blades, with twisted chiral packing of DNA, and subsequent growth along the bent blades with decreasing pitch length (see Supplementary Figure S8)^33^.

When the substrate surface was pretreated with H_2_SO_4_/H_2_O_2_, the resulting hydrophilic surface, which had a larger abundance of silanol groups, was easily chemically functionalised by TMAPS to obtain more intensive quaternary ammonium groups. As shown in Figure 2b, the long-scale DNA–silica films were composed of compactly arranged impellers with a uniform radius of ~600 nm and a thickness of ~100 nm. Although non-clear blades similar to the impeller shown in Figure 2a were observed, the cleavage of the edge of the half-blades can be clearly seen in both the top- and side-view SEM images, as indicated by the arrows. This result implies that the impeller with a large pitch length would be formed with the assembling of crowded DNA, which was subsequently confirmed by circular dichroism spectroscopy (*vide post*). The 2D-square *p*4*mm* structure and the long pitch length (~120 μm) of this film were also confirmed by HRTEM analysis (see Supplementary Figure S9). After calcination, the macroscopic helical morphologies of both films synthesised without pretreatment and with the H_2_SO_4_/H_2_O_2_ pretreatment were maintained to form pure inorganic chiral silica films (see Supplementary Figure S10).

### OA responses of the chiral films

**Figure 5:**
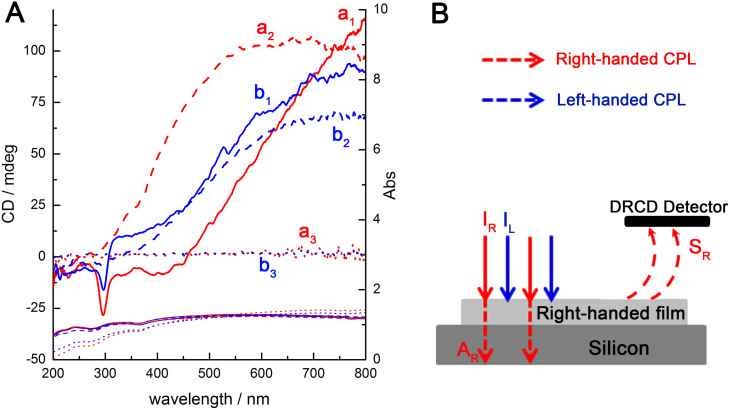
OAs of the as-prepared CDSFs and the corresponding calcined CSFs. (A) DRCD and DRUV–Vis spectra of the as-prepared (a_1_ and b_1_) CDSFs, calcined CSFs (a_2_ and b_2_) and calcined CSFs with infiltration with water (a_3_ and b_3_); the spectra showed the microscopic chirality of the DNA packing and macroscopic helical blades of the impeller. (B) Schematic illustration of the scattering-based DRCD signals of the right-handed chiral films on a silicon-wafer substrate, where *I* is the incident light (*I_L_* = *I_R_*), *A* is the absorption light, and *S* is the scattering light. For reflection DRCD, the detector signal is *D_L_* = *S_L_* = *I_L_* − *A_L_*, and *D_R_* = *S_R_* = *I_R_* − *A_R_*. The DRCD signal is CD = (*A_L_* − *A_R_*) = (*I_L_* − *S_L_*) − (*I_R_* − *S_R_*) = *S_R_* – *S_L_ = D_R_ − D_L_*. For right-handed chiral films, *S_R_* > *S_L_*. Therefore, DRCD > 0, which gives rise to positive DRCD signals.

The DNA chiral packing and impeller-like helical morphology of both the as-prepared and calcined films endowed the materials with OA, which were unambiguously detected by diffuse-reflection ultraviolet–visible (DRUV–Vis) and diffuse-reflection circular dichroism (DRCD) spectroscopy (Figure 5). The DRUV–Vis spectra of both the as-prepared CDSFs are shown in Figures 5 (a1, b1), and each of these CDSFs exhibited a broad absorption band in the range of 200 to 800 nm; these bands were attributed to the absorption of the silicon wafer due to its band gap, which is substantially greater than 1 μm^34^. Among the incident light, some would be absorbed or scattered by the films, and the transmitted parts would through the thin transparent DNA–silica films and would be totally absorbed by the silicon wafer. The absorption band of the DNA molecules and the DNA chiral packing located in 200–350 nm range overlapped with the absorption band of the silicon substrate. Each film exhibited a sharp negative DRCD signal at 295 nm and a broad positive DRCD signal in the range of 400 to 800 nm (see Supplementary Figure S11). The strong nonconservative DRCD signals at approximately 295 nm for the as-prepared CDSFs are a result of the long-range twisted arrangement of DNA layers, which corresponds to a resonance phenomenon in the absorption band of the chromophores of the long-range and highly-ordered DNA chiral mesostructures^21^,^25^,^26^. The left-handed DNA packing absorbed right-handed CPL, and reflected left-handed CPL was detected as a negative DRCD signal (see Supplementary Figure S12)^35^. The DRCD signals at approximately 295 nm completely disappeared after calcination due to a lack of chiral packing of the DNA.

However, after calcination, the DRCD signals at 400–800 nm were maintained and blue-shifted ~200 nm, which suggests that these DRCD signals originated from the light scattering of macroscopic helical morphologies of the inorganics (blades/impellers). A medium with left-handed helical morphology is known to reflect left-handed CPL and to transmit right-handed CPL, whereas the opposite is true for a medium with a right-handed chiral structure^15^. Here, for the chiral films grown on the silicon wafer substrates that absorb the full range of DRUV–Vis light, the right-handed CPL was reflected and detected as a positive DRCD signal. In addition, the left-handed CPL was transmitted and subsequently absorbed by the silicon substrate (Figure 5B). The creation of scattering-based OA originated from both the left-handed blades and the right-handed impellers (Figure 4), thereby resulting in negative and positive DRCD signals, respectively. The observed DRCD signals may be the superposition of the two opposite OAs. Therefore, the right-handed impellers would have a predominant effect on the OA of these chiral films. One impeller contains only 6–10 blades and lacks long-range orientational chirality, whereas ~800 DNA layers with long-range chiral orientation are in one blade, which would be maintained even after calcination (Figure 4). The circular Bragg reflection occurs at wavelengths *λ = Pn*_avg_/*m*, where *n*_avg_ is the average refractive index, *P* is the helical pitch length, and *m* is the order of the reflection^27^,^36^,^37^,^38^,^39^. The parameter *n_avg_* can be calculated using the relationship 1/*n_avg_*^2^ = [*l*_1_(1/*n*_1_^2^) + *l*_2_(1/*n*_2_^2^)]/(*l*_1_ + *l*_2_), where *l*_1_ and *l*_2_ are the volume fractions of components 1 and 2 that form the films. The parameter *m* is an integer value that leads to a visible-dominant wavelength (*λ*), and the OA is readily determined. The blue-shift phenomenon of the DRCD signals upon removal of the DNA templates can be explained in terms of the physical properties of the chiral medium. The average refractive indices of the DNA–silica composite films were calculated from two components (i.e., SiO_2_ (*n* = 1.46)^16^ and DNA (*n* = ~1.65, depending on the physical states^40^)), whereas that of the calcined pure inorganic silica films were obtained from SiO_2_ (*n* = 1.46) and air (*n* = 1). The change in *P* of the chiral films also induced a significant change in the scattering wavelength. When the *P* of the blades decreased (see Supplementary Figure S7), the DRCD signals attributed to DNA packing were maintained in the same peak location, whereas that of the corresponding inorganic blades were blue-shifted from the near-infrared to ~550 nm; these peaks disappeared and were maintained, respectively, after calcination (see Supplementary Figure S13), further confirming the absorption-based and scattering-based OA present in CDSFs, respectively.

The unique properties of the calcined CSFs were further examined by the absorption of water (isotropic liquid). After infiltration with water, the scattering-based OA completely disappeared (Figures 5 a3 and b3). A similar effect has been reported for engineered helical inorganic MgF_2_ films^15^ and for freestanding chiral mesoporous films^17^. In these cases, this effect was attributed to the matching of the approximate refractive index (*n*) between the water (*n* = 1.33) in the pores/media and the silica inorganic framework. Another possible reason for the OA disappearance is the presence of a water film layer on the CSFs, which scatters all of the light irrespective of the chiral medium. The intensity increase of the broad absorption band in the range of 400 to 800 nm (DRUV–Vis) provides direct evidence for the increase in the water-induced light absorption due to disappeared film scattering. These changes in the OA were exactly reversible upon drying, disappeared with the addition of water and could be repeated for more than 10 cycles (see Supplementary Figure S14).

**Figure 6:**
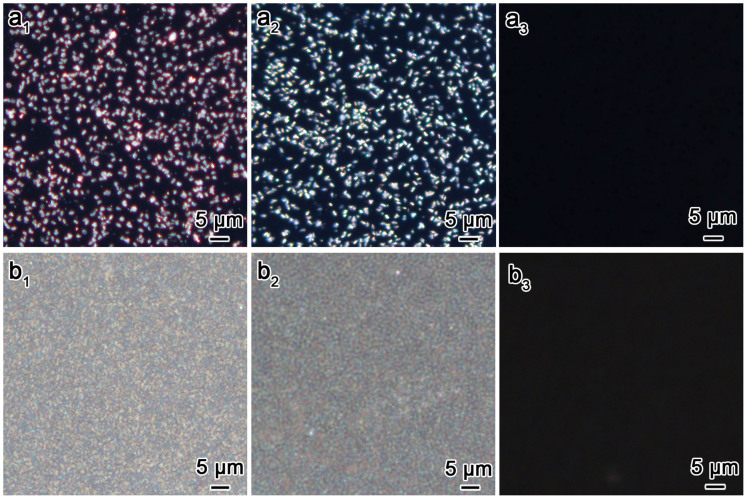
Birefringence of chiral films. POM images of the as-prepared CDSFs (a_1_, b_1_) and calcined CSFs before (a_2_, b_2_) and after (a_3_, b_3_) infiltration with water with crossed polarisers.

All of the as-prepared CDSFs and calcined CSFs are birefringent, as a result of the reflection of the scattering-matching periodic mesostructure of the films. All of the films with scattering-based OA were strongly birefringence in appearance when observed by polarised optical microscopy (POM), which further confirms the anisotropy of the helical films (Figure 6). After calcination, the films remained birefringent; however, the POM coloration changed from red to blue due to the change in *n_avg_*, as previously mentioned^17^. This result provided visible evidence for the blue shift of the DRCD signals. When water was added to the calcined CSFs, the birefringence of the materials was nearly completely terminated (Figure 6a3)^17^,^20^. Notably, the films with sparse half-impellers exhibited discontinuous dotted coloration, which indicated that the irradiance unit is an impeller. In addition, the films with the impellers arranged densely displayed uniform coloration, which indicated that real continuous chiral films can be formed via this synthesis route.

## Discussion

To the best of our knowledge, this work represents the first synthesis of an optically active inorganic chiral film via the self-assembly of DNA. Hierarchical helical structures in the CDSFs exhibited dual OAs corresponding to the DNA chiral packing and the inorganic silica helical morphology. The helicity of the CDSFs resulted in chiral reflectance regulated across the entire visible spectrum and into the near-infrared region. Such chiral materials can be used as hard templates to synthesise a variety of hierarchical helical inorganic hybrid films with OA. In addition, this simple and powerful strategy for the synthesis of chiral films has established an avenue for the production of diverse chiral inorganic films on supporting substrates using a template-based route. The helical nature of the chiral films may also find applications in catalysis, separation technology and tuneable reflective filters and sensors. Finally, the ability to switch the wide tunability of the helical pitch and the corresponding scattering-based OA combined with the facile transformation of solvent-induced absorption OA indicate that optically active chiral films may find applications in optical sensing devices.

## Methods

### Synthesis of the as-made CDSFs and calcined CSFs

The chiral DNA–silica films were grown under static conditions from a precursor solution. A typical reaction mixture consisted of 10 mg of DNA (Herring tests, Sigma-Aldrich), 8.4 mg of MgCl_2_·6H_2_O (Sinopharm Chemical Reagent Co. Ltd.), 100 μL of TMAPS (50% in methanol, TCI) and 100 μL of TEOS (99%, Sigma-Aldrich). This amount of Mg^2+^ corresponds to an optimal Mg^2+^/DNA ratio of 1.5, which we determined after studying the influence of the molar Mg^2+^/DNA ratio in the range of 0–2, where much higher and lower ratios gave rise to less-ordered and perfect CDSFs and even non-helical impellers on the supporting substrate. TMPAS and TEOS were stirred for 30 min for prehydrolysis. The silicon wafer was added to the TMAPS solution (2.5 mL TMAPS/200 mL toluene) under reflux for 4 h to obtain the quaternary-ammonium-functionalised surface. The silicon wafer was subsequently immersed in the DNA solution (ultrasonically treated) for 30 min, and then Mg^2+^ and TMAPS/TEOS (prehydrolysis) were successively added with oscillation such that the CDSF were grown on the surface of silicon substrate. The pH value of the reaction solution was adjusted with HCl and NaOH. The mixture was then allowed to react under static conditions at 25°C for 1 day. The as-prepared CDSFs were washed twice with ethanol and deionised water to remove the unreacted silica source and powder. Finally, all of the organics in the CDSFs were removed by calcination at 550°C to form the calcined CSFs. The calcined CSFs with absorbed water were prepared by soaking the films in water and were tested directly for DRCD.

### Characterization

The microscopic features of the samples were observed through SEM (JEOL JSM-7401F). For the TEM observations, the samples were scraped from the chiral film, and dispersed in ethanol and dropped on a carbon thin film on a Cu grid. HRTEM observations were performed with a JEOL JEM-2100 microscope operating at 200 kV. DRUV/Vis and DRCD spectra were collected using a JASCO J-815 spectropolarimeter equipped with a DRCD apparatus. Note that all the present DRCD sample spectra were obtained by averaging the signals at different angles by rotating the sample in order to disregard the effect of linear birefringence and linear dichroism.

## Author Contributions

S.C. contributed to the design of the experiments and guided all aspects of the work. B.L. synthesised the chiral films and performed the SEM and CD measurements. L.H. processed the HRTEM characterisation and structural analyses. S.C., L.B., Y.D., Y.C., J.F. and Y.Y. contributed to the CD analyses. S.C. and B.L. prepared the manuscript and contributed to the analysis of the mechanism.

## Supplementary Material

Supplementary Information Supplementary Informations

## Figures and Tables

**Figure 1:**
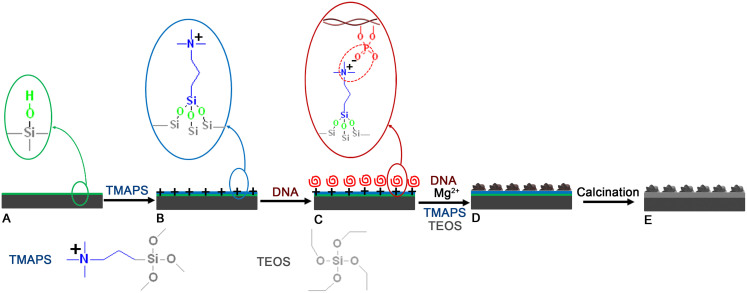
Schematic representation of the formation of CSFs. (A) The surface of the substrate is treated to create abundant silanol groups, which can be rationally controlled with the pretreatment method. (B) The quaternary ammonium groups are chemically modified on the substrate surface by co-condensation between the silanol and siloxane of TMAPS. (C) Parallel-aligned DNA molecules are arranged on the surface by electrostatic interaction between the quaternary ammonium groups of the substrate and the phosphate groups of DNA. (D) The formation of CDSFs is due to chiral DNA packing, and the CDSFs are subsequently arranged on the surface of the substrate by the self-assembly of DNA, TMAPS and TEOS in the presence of Mg^2+^. (E) The pure CSFs are obtained by calcination to remove the DNA.
