# Supplementary material for: Growth of Optically Active Chiral Inorganic Films through DNA Self-Assembly and Silica Mineralisation
Source: Sci Rep. 2014 May 2;4:4866. doi: 10.1038/srep04866 (PMC4007082; doi:10.1038/srep04866)
Supplement: Supplementary Information — s [file srep04866-s1.doc]

**Supplementary Information**

**Growth of Optically Active Chiral Inorganic Films through DNA Self-Assembly and Silica Mineralisation**

**Ben Liu, Lu Han, Yingying Duan, Yunayuan Cao, Ji Feng, Yuan Yao and Shunai Che***

School of Chemistry and Chemical Engineering, State Key Laboratory of Metal Matrix Composites, Shanghai Jiao Tong University, 800 Dongchuan Road, Shanghai, 200240, China

*e-mail: [chesa@sjtu.edu.cn](mailto:chesa@sjtu.edu.cn)

**
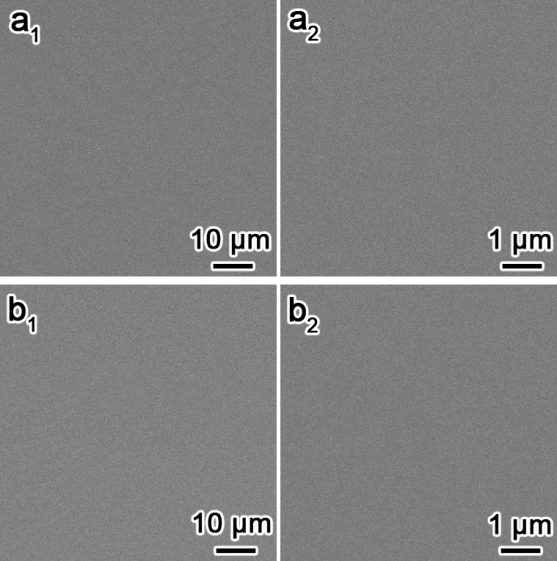
**

**Supplementary Figure S1.** Low- and high-magnification SEM images of the silicon substrates without pretreatment (a) and with H_2_SO_4_/H_2_O_2_ pretreatment (b).


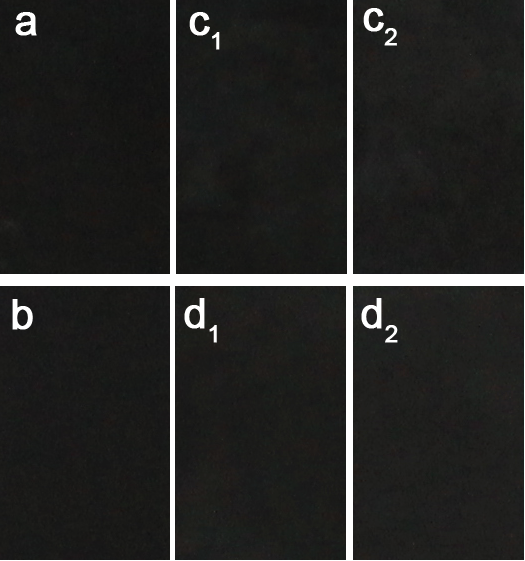


**Supplementary Figure S2.** **Photographs of the silicon substrate, as-prepared CDSFs and calcined CSFs.** a) A silicon wafer; b) a calcined silicon wafer; c_1_) and c_2_) as-prepared CHDSF and calcined CSF without pretreatment, as shown in Figure 2a; d_1_) and d_2_) as-prepared CDSF and calcined CSF with H_2_SO_4_/H_2_O_2_ pretreatment, as shown in Figure 2b.


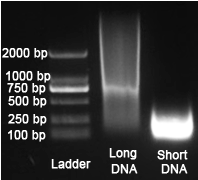


**Supplementary Figure S3. The 2% agarose gel electrophoretic separation of DNAs and sonicated DNAs.** Lane 0, DL2000 ladder; lane 1, type XIV DNA sodium salt from herring tests from Sigma-Aldrich; lane 2, sonicated type XIV DNA sodium salt.


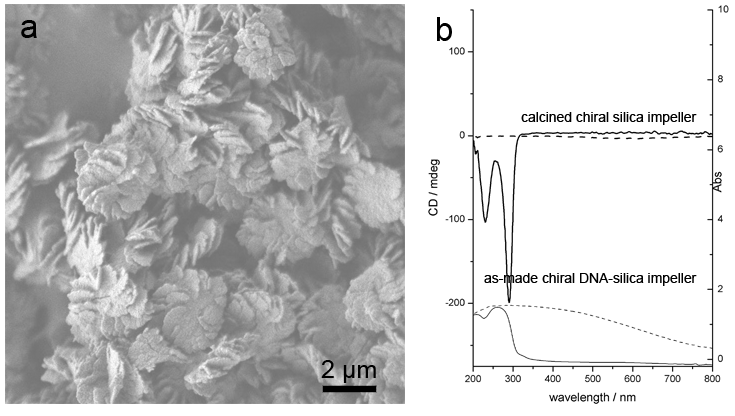


**Supplementary Figure S4. Macroscopic helical morphology and corresponding OA of chiral DNA-silica impeller powder.** As-made chiral DNA-silica impeller exhibited two strong CD signals at around 295 and 230 nm, indicating the long-range chiral DNA arrangement. No CD signals can be found in the range of 400-800 nm, suggesting that the powder sample was lack of scattering-based OA, which has been further confirmed by CD signals of calcined chrial silica impellers.


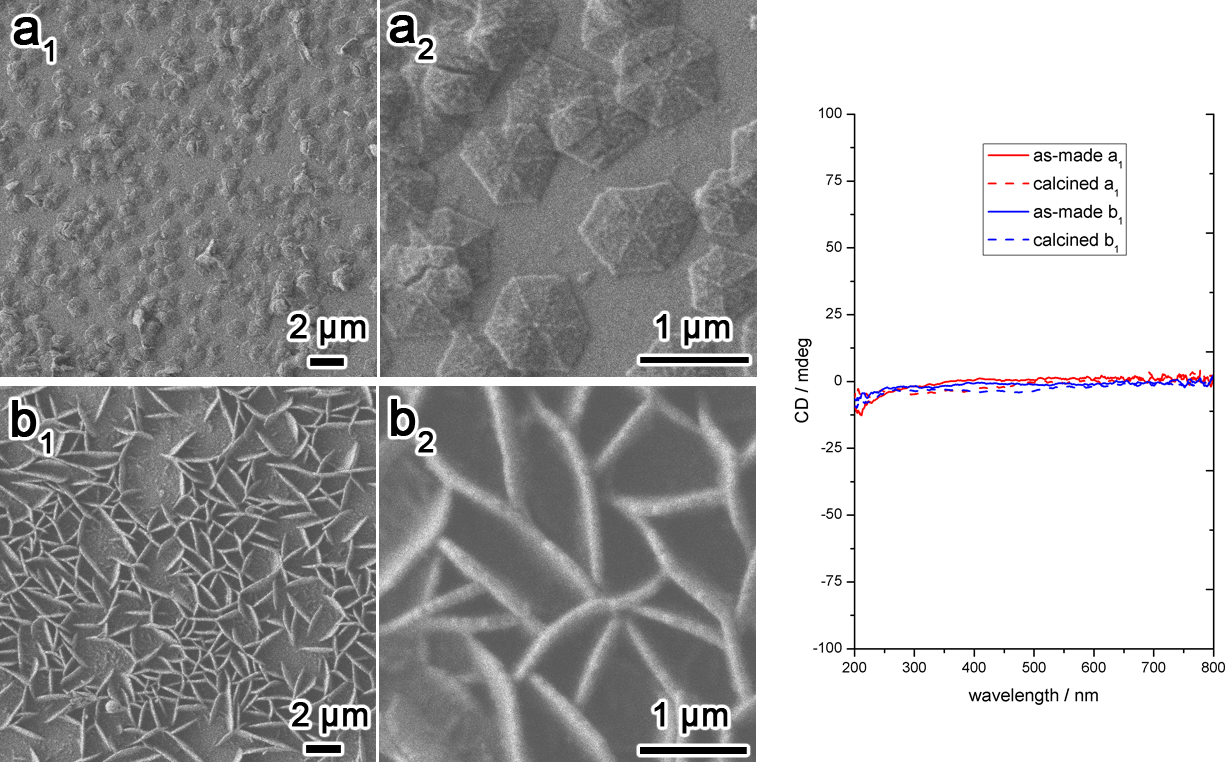


**Supplementary Figure S5. Macroscopic morphology and corresponding OA of horizontally (a) and vertically (b) aligned DNA-silica films.** Both the films with DNA-silica platelet-like morphology (not impeller-like helical morphologies) are lack of CD signals, indicating optical activity can be only exhibited by chiral films


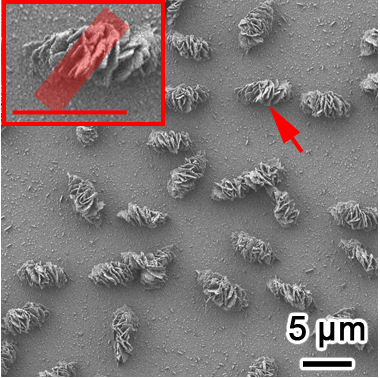


**Supplementary Figure S6. Macroscopic helical morphology of the CDSF.** The IHDSC with blades arranged in a clockwise manner in the side view (i.e., at an inclination angle smaller than 90° from left-bottom to right-top from the top view as shown insert in Figure S6) is defined as left-handed.


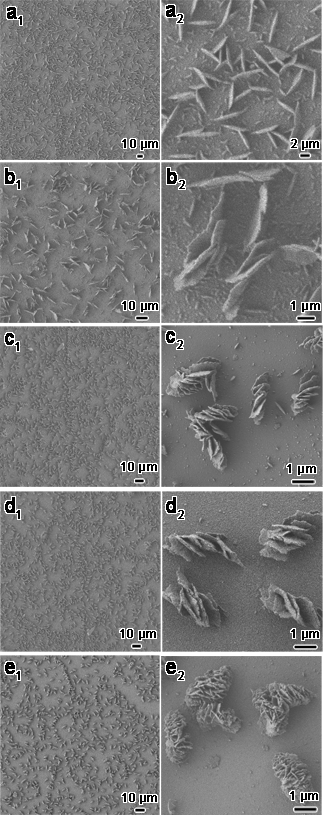


**Supplementary Figure S7.** SEM images of the as-prepared CDSFs with different inclination angles synthesised with different Mg^2+^/DNA molar ratios. The synthesis molar composition of DNA/Mg^2+^/TMAPS/TEOS/H_2_O is 1/x/6/15/18,000, where x = 0 (a), 0.5 (b), 1 (c), 1.5 (d, as shown in Figure 2a) and 2 (e).


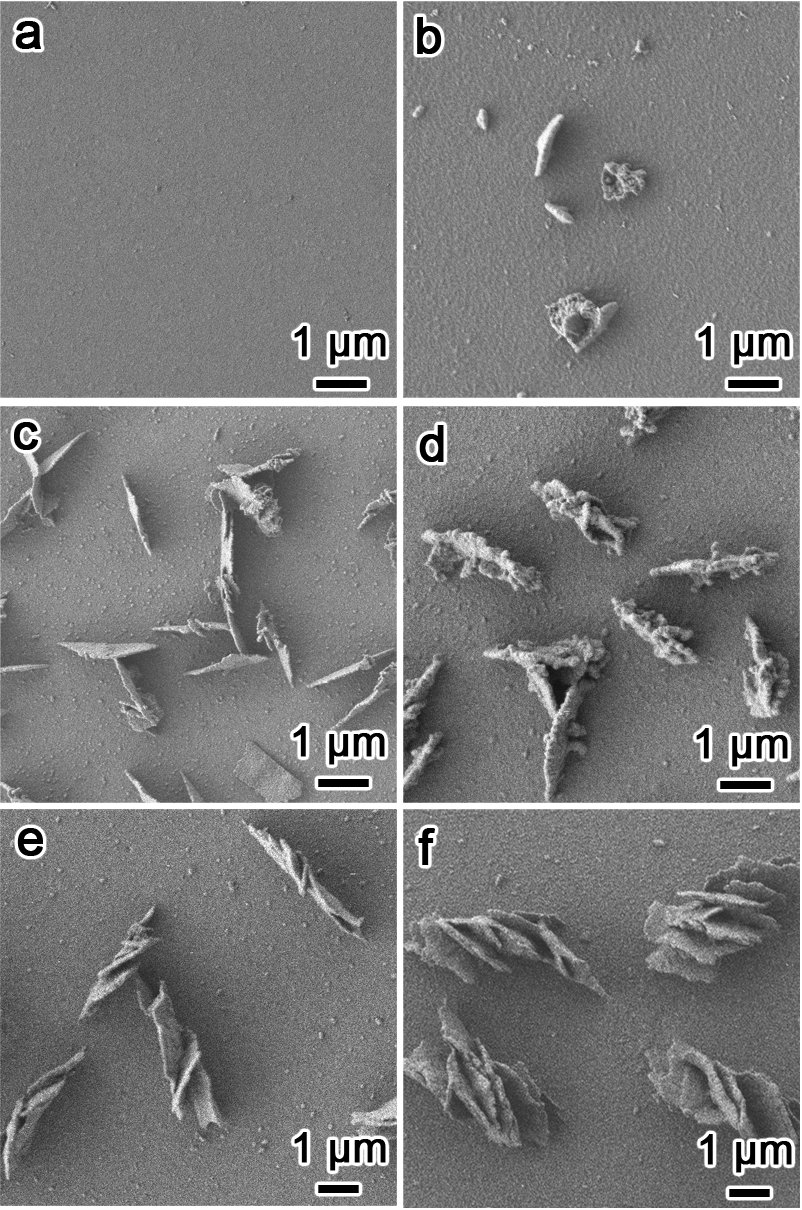


**Supplementary Figure S8.** Morphological transformation of the CDSFs after different reaction periods. The reaction time is 1 h (a), 2 h (b), 3 h (c), 5 h (d), 8 h (d) and 1 day, respectively. Initially smooth sheets (b) were transformed into the impeller-like helical morphology with a number of distinct lobes (c, d) through the breakage of the edges into multiple blades (e) and the subsequent curving of the bent blades (f).


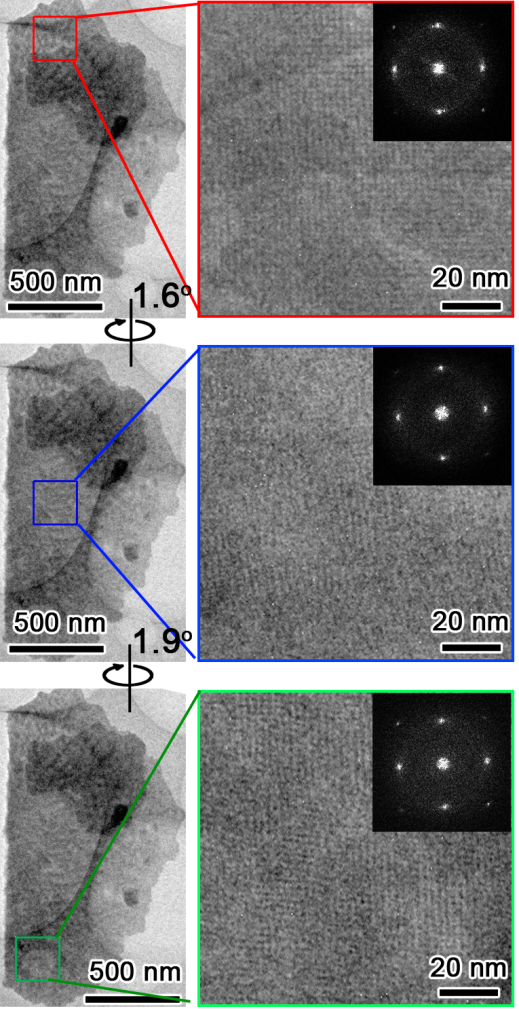


**Supplementary Figure S9.** SEM images of the CDSFs with pretreatment of H_2_SO_4_/H_2_O_2_, as shown in Figure 2b. The 2D-square contrast was aligned in the middle and bottom portions by clockwise tilting of the blades along the (10) axis by 1.6° and 1.9°, which indicated that the twisted DNA columnar packing in the CDSFs is left-handed (Figure 4). The distance from the top to the middle potions (0-1.6°) and from the middle to the bottom portions (1.6-3.5°) is ~0.61 and ~0.58 μm, respectively. Therefore, the average pitch length of the blades was calculated as (0.61 + 0.58 μm) × 360°/ 3.5° = 122.4 μm.


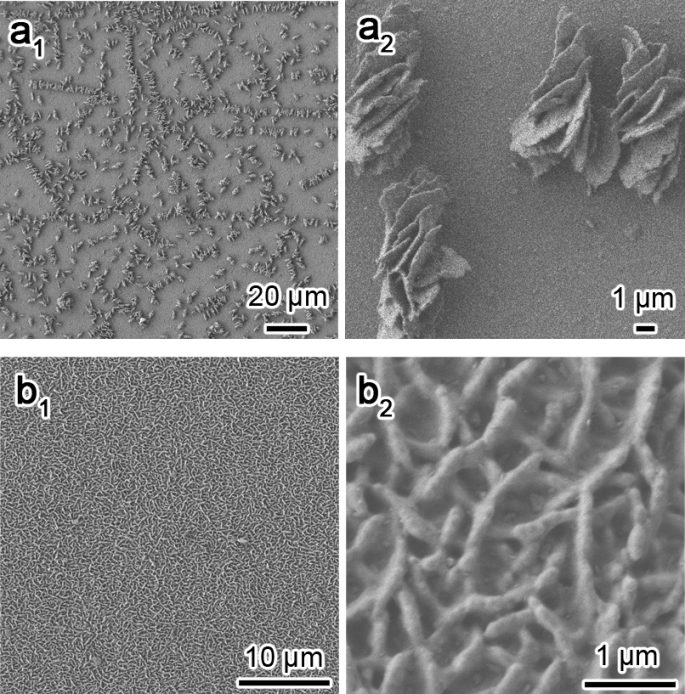


**Supplementary Figure S10.** SEM images of the calcined CSFs shown in Figure 2.

**
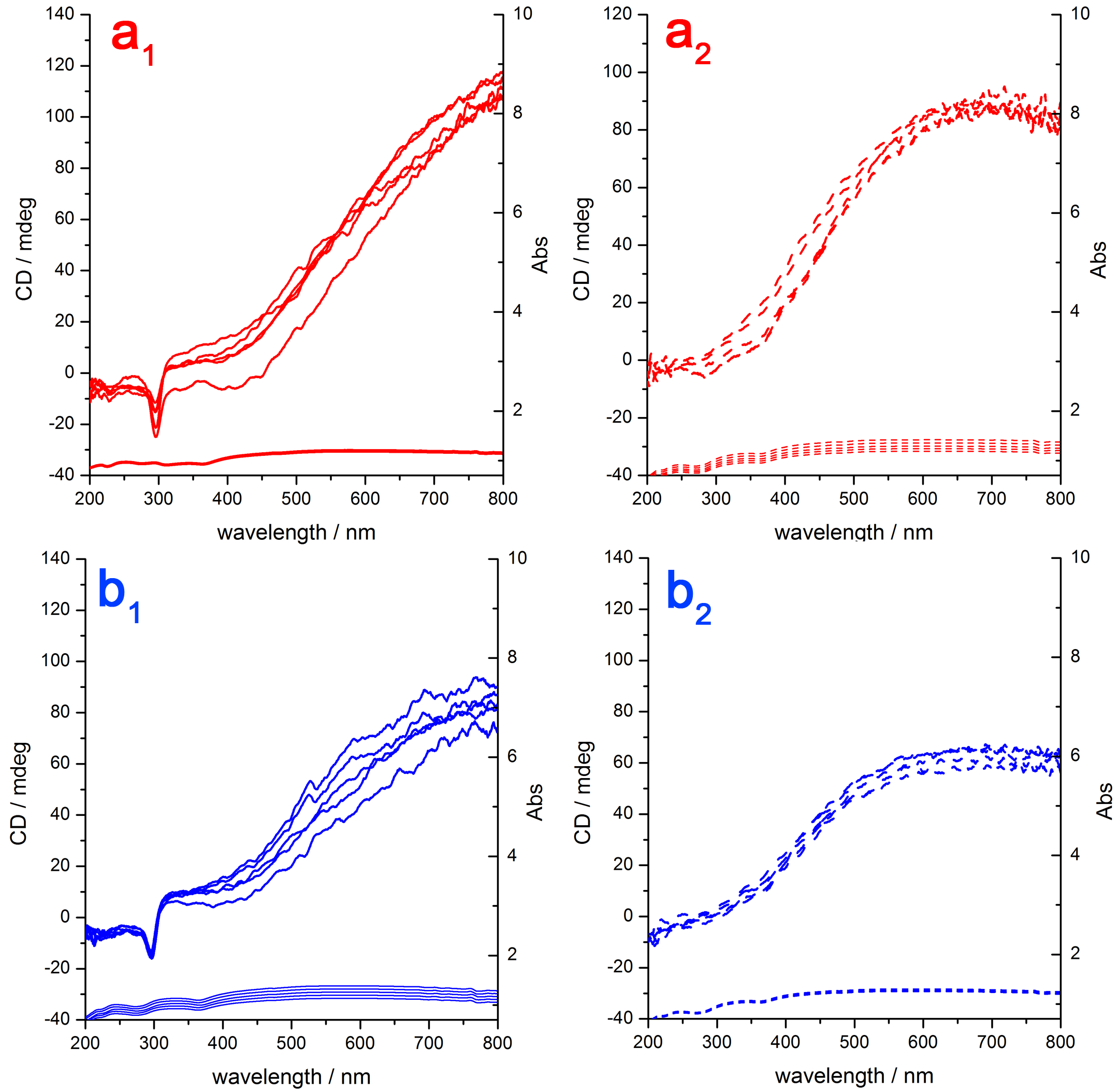
**

**Supplementary Figure S11.** DRCD and DRUV–Vis spectra of the as-prepared (a_1_ and b_1_) CDSFs and calcined CSFs (a_2_ and b_2_) calcined CSFs shown in Figure 5 at different angles (0°, 45°, 90°, 135°, 180°) by rotating the sample. It should be noted that all the present DRCD sample spectra shown in Figure 5 and afterward were obtained by averaging the signals at different angles by rotating the sample in order to disregard the effect of linear birefringence and linear dichroism.

**
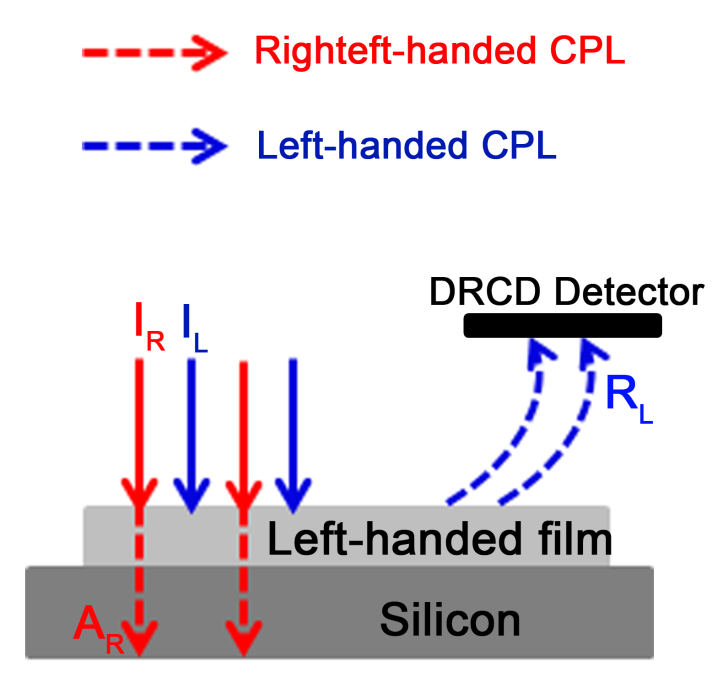
**

**Supplementary Figure S12.** DRCD signals of the chiral DNA packing in CDSF induced by the absorption of CPL, where *I* is the incident light (*I_L_* = *I_R_*), *A* is the absorption light, and *R* is the reflection light. The absorption DRCD signal is calculated as DRCD = *A_L_*–*A_R_* = (*I_L_*-*S_L_*)-(*I_R_*-*S_R_*) = *S_R_*–*S_L_ = D_R_-D_L。_* For left-handed chiral films, *S_L_ > S_R_*. Therefore, DRCD < 0, which gives rise to negative DRCD signals.


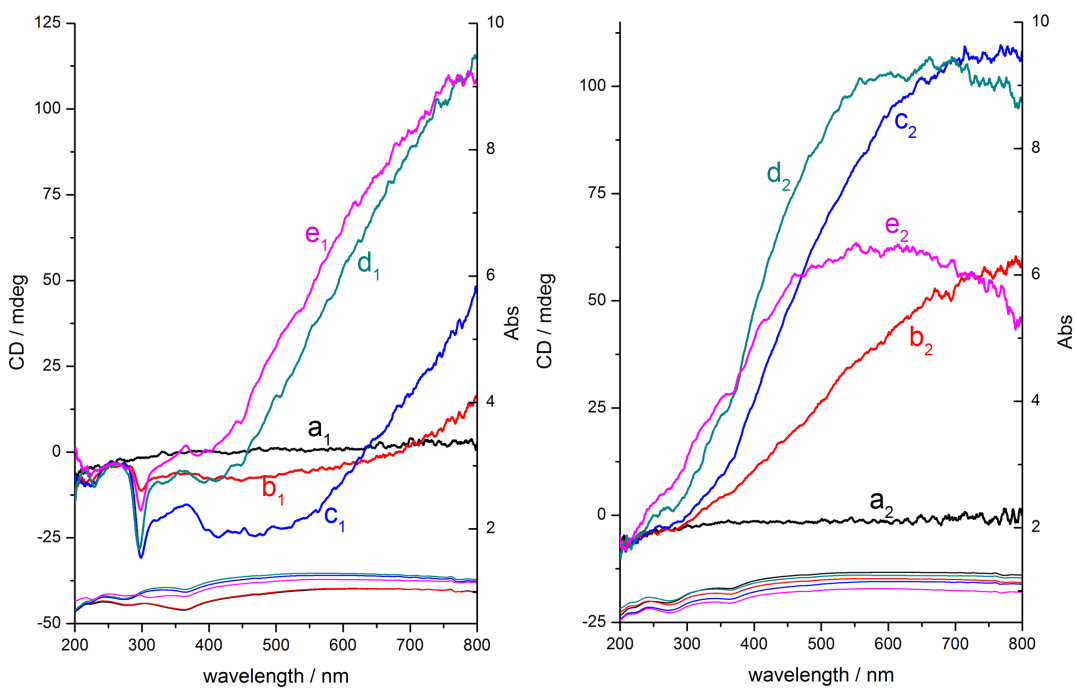


**Supplementary Figure S13.** DRCD and DRUV–Vis spectra of the as-prepared CDSFs (left) and calcined CSFs (right) shown in Supplementary Figure S7.


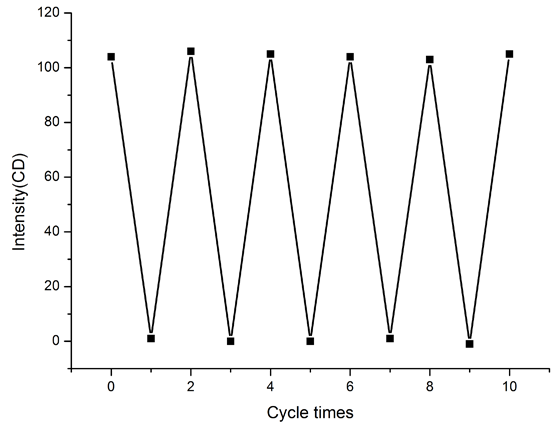


**Supplementary Figure S14.** Cycling graphs represent DRCD maxima exhibited by the calcined CSFs before and after the infiltration of water.
